# Clinical Strategies to Develop Connections, Promote Health and Address Pain From the Perspectives of Indigenous Youth, Elders, and Clinicians

**DOI:** 10.3389/fpain.2022.857624

**Published:** 2022-05-13

**Authors:** Rachel VanEvery, Margot Latimer, Angela Naveau

**Affiliations:** ^1^Department of Health, Aging, and Society, McMaster University, Hamilton, ON, Canada; ^2^Faculties of Health and Medicine, Dalhousie University, Centre for Pediatric Pain, IWK Health, Halifax, NS, Canada; ^3^De dwa da dehs nye>s Aboriginal Health Centre, Hamilton, ON, Canada

**Keywords:** Indigenous youth, clinical strategies, Two-Eyed Seeing, pain, hurt, community led research

## Abstract

In this article we discuss findings from a community based, participatory action research study. The aim was to understand how Indigenous youth describe, experience, manage pain and hurt and how they seek care. A critical analysis guided by Two-Eyed Seeing and Medicine Wheel frameworks highlighted important clinical strategies for Indigenous youth to balance their health and reduce pain. This study is a partnership project with an Aboriginal Health Centre in Southern Ontario and the Canadian Institute of Health Research funded Aboriginal Children's Hurt and Healing Initiative (ACHH). The study gathered perspectives of Indigenous youth, Elders, and health clinicians using conversation sessions guided by a First Nations doctoral student and nurse researcher. Using the medicine wheel framework three main thematic areas emerged across the three groups and include (1) Predictors of Imbalance; (2) Indicators of Imbalance; and (3) Strategies to re-establish balance health in relation to pain. The main strategy includes considerations for clinicians using the acronym LISTEN (*Language, Individual, Share, Teachable moments, Engage, and Navigate*) approach that outlines strategies for clinicians that will be a safe guide to manage pain and hurt.

## Introduction

Understanding how Indigenous youth express hurt and pain in a clinical setting is critical for promoting a healthy life trajectory, including mental, emotional, spiritual, and physical wellbeing (1). Developing strategies to support Indigenous youth during this time is critical to healthy development and promoting positive experiences with health care providers. In particular, the impact of culturally safe and Indigenous-led health care has been demonstrated to improve health outcomes, integrate Western and Indigenous knowledge in care plans, and improve access and adherence (2). Indigenous Peoples in Canada experience high rates of physical pain compared to the non-Indigenous population, and the primary conditions for seeking health care relate to the following pain related symptoms; headaches, musculoskeletal, oral and ear that can profoundly impact children's growth and development (3, 4). The loss of Indigenous knowledge has disrupted Indigenous health systems, leaving few resources for youth to use to conceptualize pain and its associated health outcomes.

In 2016, approximately 26.8% of Indigenous Peoples in Canada were 14 years of age or younger, with the largest population living in the province of Ontario (5). There is a growing body of literature about the context of Indigenous children and youth's experience with pain, including a learned stoic expression of pain and hurt (1, 6–9) that may be impacting clinician's ability to accurately assess and subsequently manage that pain. Additionally, there are misunderstandings about the impact of historical trauma as a driver of pain and hurt that has led to persistent stereotypes. Two Eyed-Seeing approaches to clinical care and an indication of the importance of creating safe spaces to share pain have been identified as important considerations for health systems and clinicians (1, 4, 10). Creating safe health care spaces to engage Indigenous youth in meaningful hurt and pain conversations is an important way to promote health and mitigate the harmful impacts of intergenerational trauma. Indigenous people in Canada experience higher rates of adverse health outcomes and lower rates of specialist care referrals compounded by inequitable access to health care (4).

Untreated hurt and pain negatively influences participation, physical activity, academic performance, language and social development, sleep patterns, growth, behavior, mental health and substance use, therefore burdening future choices concerning their lives and health care utilization (11–16). In one study with First Nations people in Mi'kma'ki, participants identified the importance of considering who *family is to Indigenous People*, how *information* is exchanged between patient and clinician, building *relationships*, creating *safe spaces*, and considering *treatment* from both an Indigenous and western lens when supporting Indigenous people in the healthcare context (1). Recognizing the negative historical impact on Indigenous Peoples care seeking behaviors, lack of trust with the health care system and the reluctance to express themselves in terms of pain means prioritizing Indigenous youth and supporting them to navigate the healthcare system more effectively. Health clinicians can better support Indigenous youth to confidently express pain within clinical settings to meet their needs and address barriers for better health outcomes.

The negative effects of untreated pain can disrupt growth, sleep patterns, language development, social development, and mental health (17, 18). Youth with familial ties to Indian Residential Schools are at a higher risk of experiencing psychological pain and distress, suicidal thoughts, trauma, mental health issues, and physical pain (19–21). As a result of a long history of forced assimilation and colonization, significant inequalities exist for Indigenous youth. Therefore there is an important role for clinicians and Elders in the institutional health care system to improve health outcomes (22).

There is little evidence related to how urban Indigenous youth engage while in a clinical setting and describe pain and hurt as it relates to their health and overall wellbeing. The aim of this study is to mobilize Indigenous knowledge to improve the health care experiences for urban Indigenous youth. This study is an extension of the Canadian Institute of Health Research Chronic Pain Network (CPN) funded Aboriginal Children's Hurt and Healing (ACHH) Initiative, originating in Mi'kma'ki. This project, consistent with the original research conducted in Mi'kma'ki, is Indigenous community led, implemented with the knowledge interpreted and disseminated by community members with the process described in work by Sylliboy et al. (23). The larger study explored the expression of pain through art as well as healthcare utilization data with Indigenous children and youth in First Nations communities in Nova Scotia, New Brunswick and Prince Edward Island (1, 4, 10).

## Methods

### Two-Eyed Seeing Approach

A Two-Eyed Seeing approach, coined by Mi'kmaw Elders Albert and Murdena Marshall, indicates both Indigenous and Western knowledge should be considered equally beneficial to co-create new learning's relevant to support the health and wellbeing of Indigenous peoples (24). Two-Eyed Seeing (TES) originates and mirrors how Indigenous people have evolved in their way of living in their ancestral territories, adapting to the evolving circumstances given the impacts of colonialism. TES considers the strengths and perspectives for every aspect of the research process (question development, engagement, methods, data gathering, interpretation and dissemination). The Two-eyed Seeing approach was utilized as a guiding principle to engage, gather, and interpret knowledge in this study. The first author (VanEvery) was the Research Coordinator in this study and contributed her lived experiences and Indigenous knowledge throughout the research process. She is Grand River Mohawk and has degrees in both nursing and public health. The second author (Latimer) is a settler Canadian with Scottish ancestry and has been working with Indigenous communities to mobilize community knowledge to improve health experiences. The third author (Naveau) is Anishnaabe Kwe, Bear Clan and a member of Mattagami First Nation in Northern Ontario. She began her journey with the Aboriginal Health Centre in 2004 and is dedicated to aligning Indigenous practices and ways of knowing and being to enhance the healthcare experiences and relationships for Indigenous people. Throughout the application of the Two-Eyed Seeing approach, data analysis was completed independently and then collectively by all authors. The authors met virtually over the period of several months, followed by an in-person visit where the ACHH team came together to discuss results and to clarify interpretation of the data, so that consensus could be reached. Using both Indigenous and Western perspectives, the data was interpreted to bridge both knowledge systems and to help clinicians understand how Indigenous youth express, share, and manage their pain.

### Community Engagement

In keeping with the TES approach described by Sylliboy et al. (23) and to determine if the study goals would benefit and fit with the community expectations, a pre-study community engagement session took place with ACHH researchers and two health care providers, two Elders, two youth, as well as the Aboriginal Health Centre's privacy officer and a Nurse Research Coordinator. This study employed a community based, participatory action research partnership with an Aboriginal Health Centre in Ontario and the ACHH Initiative research team following the Ownership, Control, Access, and Possession (OCAP) principles (25). The ACHH Initiative aims to work with communities, clinicians and universities to share knowledge that will improve Indigenous child and youth wellness by mobilizing Indigenous knowledge gathered and owned by community, into the health system.

This qualitative study sought to understand how Indigenous youth describe, experience, and manage pain and hurt and how they seek care. Youth, Elders, and health clinicians selected one-on-one interviews or small group conversation sessions and each session lasted approximately 1 hour. The research protocol received approval from an Indigenous Ethics Review Board and the partnering pediatric health care setting. A contract was signed by each of the two parties outlining the community ownership of the knowledge gathered and all decision-making was at the discretion of the community.

### Setting and Sample

The setting of this study took place in two urban communities in Southern Ontario, Canada where both cities are located on the Iroquois Territory. There are several terms used to refer to Indigenous or Aboriginal people or in the US Native Americans. In this study we recognize Indigenous or Aboriginal people who in Canada, comprise of the First Nations, Inuit and Metis.

The Aboriginal Health Centre is available to self-identifying Indigenous peoples living in Hamilton, Ontario with an estimated population of 12,130 and Brantford with a population of 5,395 Indigenous people (26). The Aboriginal Health Centre serves to improve the wellness of Indigenous people and the Indigenous community by offering a blend of primary care and traditional healing services with respect to everyone's distinct cultural identity, values and beliefs. The Aboriginal Health Centre offers youth an Aboriginal Youth Mental Health Patient Navigator to ensure that the individualized needs of youths are addressed with appropriate and culturally safe support. This role has been crucial in helping youth reconnect within their communities; especially those involved in the Child Welfare system. This service aids in the development and guidance for youth to engage in a support system similar to a traditional family unit. The Aboriginal Health Centre also offers traditional healing guided by the principles of the Medicine Wheel to promote spiritual and emotional wellness. Registered patients have access to both traditional healers and Western medicine primary care clinicians. Traditional teachings are offered by Elders, knowledge keepers and medicine people. There is also access to cultural ceremonies and community events, workshops promoting cultural arts and ways of knowing and being, as well as one-on-one peer emotional support and hands on healing bodywork modalities.

There were three groups of participants: Elders, health clinicians and youth. Participants were recruited by the Nurse Research Coordinator engaging in convenience sampling, including by word of mouth and recruitment posters in local Indigenous centers and gathering spaces where potential participants frequent. Additionally, the Research Coordinator was invited to youth programming at the regional Indigenous Friendship Centre. Youth participants who met the following inclusion criteria were invited to participate: (1) self-identified Indigenous identity, (2) 13 years of age and older; and (3) living in Hamilton or Brantford. Elders/knowledge holders and health care clinicians were invited to participate if they had experience working with Indigenous youth in this area. They were recruited by email invitation, poster invites, and by word of mouth. The Research Coordinator contacted all eligible participants *via* telephone and/or email to further explain the study, confirm their eligibility and ensure all potential participants have an opportunity to ask any questions. All participants signed written consent forms and were provided with a gift card to thank them for their participation and contribution to this study. All consents and data from this study were locked in a cabinet at the Aboriginal Health Centre with copies kept confidential at the partnering ACHH Initiative space in Nova Scotia. The community ownership and storage of their own data was in keeping with ethical approach and consistent with the Ownership, Control, Access and Possession principles (OCAP) (25).

### Data Collection and Analysis

Four sources of data were collected including: (1) Health Surveys, (2) Conversation Sessions, (3) Artwork, and (4) Child and Youth Health Centre electronic medical record (EMR) data between 2015 and 2019. This paper will share the main qualitative results from the conversation sessions with Elders, clinicians and youth. The four dimensions of the Medicine Wheel were used in the interpretation of the knowledge shared by these groups.

#### Medicine Wheel

Many Indigenous people live by the principles of the medicine wheel and it is integrally embedded in health beliefs representing the dimensions of health and healing. The circle represents the interconnectivity of all aspects of health. The Medicine Wheel is meant to convey wholeness and balance in a continuum, and the quadrants represent the 4 dimensions of well-being: physical, mental, emotional and spiritual (27, 28). The Medicine Wheel has no boundaries between the dimensions and is recognized by most Indigenous people in North America to represent the natural order or balance within the circle of life (29). The figure of the Medicine Wheel outlines each dimension and symbolizes balance, connectedness, wholeness and relationships. The dimensions of the Medicine wheel (emotional, spiritual, physical, mental) were used, as a proxy measure of the types of pain participants might be experiencing so were considered when analyzing the knowledge gathered in the conversation sessions. In related pain research (1), Indigenous study participants consistently responded that pain and hurt were experienced within the four dimensions of the Indigenous health perspective: mental, spiritual, physical, and emotional. Therefore, each dimension is used as a thematic area.

## Results

### Demographics

In total, there were 33 participants in this study, 21 were youth (13-19 y/o), 6 Elders/ Knowledge holders and 6 clinicians (Nurses, Mental Health and Social workers) resulting in 33 conversation sessions. All Elders/ Knowledge holders self- identified as First Nation, no other demographic data was collected from these participants.

### Conversation Sessions

Conversation sessions were held at the Indigenous Friendship Centre, where youth felt comfortable and safe and at a time that was convenient for the youth. Several questions were asked of the participants such as “*what does pain and hurt mean to you,” “how do youth express their pain and hurt,” “if a new doctor, nurse, or dentist was coming to work at the health centre and you had to tell them about how youth from your area express their hurt-what would you tell them.”* Data analysis using the four dimensions of the Medicine Wheel and thematic coding strategies coincided collaboratively to describe *what pain and hurt means*. One Elder said “I buried my emotional pain, so it's just physical pain that I really react to, so that's what pain means to me, just physical pain.” A clinician commented “If you go kind of based on the teachings it can also be spiritual pain where people have been deprived of their teachings” and yet another commented “hurt can be from like I said, bullying or shaming, just more emotional.” One youth said “Pain means depression” and “feeling worthless like your nothing and feeling in a really low place.” One youth described pain and hurt as both a physical experience such as scrape your knee, whereas emotional and mental pain as “something that's painful you don't want to deal with, you just wish it was over. It makes you kind of want to just die and be alone and stuff.” She went on to say, “…feels like you're trapped there in that place of pain. It makes you just want to kill yourself and have it be over.”

In response to the question “*how do youth express their pain and hurt,”* participants said, “Kids don't know their language so can't describe. Doctors use scale sometimes and you just feel dumb.” One clinician said, “I see a lot of it being held within, and that is how a lot of them were raised to deal with it, to just keep it inside. Don't show it as weakness, and crying is weakness and all of that stuff. So, I think a lot of the emotional stuff they tend to try to not acknowledge it or deal with the pain the best they can.”

There was a common theme that females were more open about expressing their emotional pain, for example a youth shared, “females hold it in and let out emotionally” but there were also general comments that they try not to cry- because one youth said, “younger siblings look up to me so I try to keep it together.” There was an understanding that “Men don't show, women do, easier for women to express their feelings.” Clinicians said youth express their pain and hurt through silence, isolation, acting out, get really moody or just pick on somebody else, perpetuate hurt toward others, and feel sorry for themselves. One Elder said “There's something missing, and that's why they go into addiction, why they have unhealthy behaviors, why they're taking medication, is because they're missing something in their life.”

When considering the notion that health is a balance of the dimensions of the Medicine Wheel three main thematic areas informed by the conversation session content emerged across the three groups (Elders, youth and clinicians) and include: (24) predictors of health imbalance, (2) indicators of health imbalance and (3) Strategies to re-establish and/or maintain healthy balance.

1. *Predictors of Imbalance*: Circumstances that *predict imbalance of health* included access to resources such as safe surroundings, stable housing, family supports and financial stability. For example, one clinician said, “Poverty is a huge contributor to mental health” while another youth commented, “violence is second nature” and as is “homelessness.”
2. *Indicators of Imbalance*: Quotes included content related to children and youth isolating themselves, bullying or being bullied, keeping the hurt inside, not attending school, hurting themselves, cutting, drinking, drugs, smoking, and engaging in risky behaviors. Youth commented “kids being bullies, and can't regulate emotions,” “I just keep it to myself until something else comes up, another argument or maybe a bad thing that pops into my head, keeping it to myself until it explodes” with one clinician saying: “I see a lot of drug use and alcohol abuse to try to cope with those traumas and pain” and another said “times are different now because of technology too, and really seeing that kids are on technology all the time. And really try to say, you know, losing touch with that human one-to-one age, right?”
3. *Strategies to re-establish balance were conceptualized using the conversation content and represented with the acronym LISTEN*. The three groups suggested ways clinicians could improve clinical experiences for Indigenous youth. The LISTEN approach includes (a) Language; (b) Individual; (c) Share; (d) Teachable Moments; (e) Engage; and (f) Navigate.

#### Language

The conversation session content that represented youth's experiences of pain, resulted in a common theme of *language*, with three subthemes as key communication barriers. Participants described three strategies for youth to build a language with clinicians, including translating healthcare terminology, exploring health literacy, and being aware of cultural body language.

##### Translating Healthcare Terminology

Youth described the language of clinical assessments as unhelpful, frustrating, and numerical. One youth participant explained how clinicians can translate healthcare terminology to support her: “I'll go to the doctor and I'll describe pain and they'll be like, what does that mean? I'm like, I don't know exactly, that's just the way I describe it, and they should be more open to it.” Another youth suggested: “Accepting your different vocabulary. I know personally I describe things very weird, or I don't know how to describe some things. It's a lot easier if they're helping you come up with a way of describing pain.”

Elders and clinicians suggested translating healthcare terminology when working with youth as they have their own language and tools that they bring with them to the clinical setting. A clinician described the barriers of quantifying assessments with Indigenous patients as follows: “that number scale doesn't really work very well...it's more telling the story of why and what the thought is around it [pain].” Failing to translate medical terminology in lay terms resulted in pain left untreated and youth coping with symptoms on their own.

##### Exploring Health Literacy

Language was described by participants in respect to the health literacy of youth. Low health literacy was prohibitive for youth who prefer to discuss their health concerns as a result of social inequities. One youth described her frustrations:

How do you even tell your doctor, oh yeah, I'm sad because I'm poor. How do you say that? Even if you were to say something like, I feel like this is something that's contributing to my poor mental health, it's like, how is your doctor going to prescribe something to fix that?

Clinicians recognized how important it was to take the time to engage in the youth's social being and when they critically examined the use of their language they could provide better pain management. One clinician suggested sensitive communication strategies and a strengths-based approach to encourage youth to address their health concerns. Furthermore, when health literacy was explored avenues were created that allowed for clinical exploration of the social determinants of health, while also building language and empowering youth to feel heard.

##### Cultural Body Language

The three groups commented about varying cultural differences in body language and expression of pain. Clinicians shared their need to assess youth beyond physical pain and more holistically in terms of identifying cultural traits. A clinician shared her suggestions: “Pain is not always sadness. The pain is not always a facial feature. Pain is not always a gesture. And you have to really dig to uncover that piece, and how it's manifesting, and not just taken for face value.” Elders discuss cultural body language of stoicism and silence as a cultural trait. However youth describe the same term as “bravery,” “masking the pain” and inability to “express hurt and pain in words.” Elders, clinicians and youth highlighted the importance of communication with youth by learning the local language, literacy and social skills of the urban Indigenous community as a way to establish balance.

#### Individual

Participants discussed balancing the unique needs of the Indigenous *individual* and role of the *institution*. The three groups shared how youth individually experience pain and hurt as a result of colonization, disconnection to culture, dysfunctional family, community, and historical trauma. One youth described her pain linking to childhood when asked, *what words do you use to describe pain or hurt to your doctor, nurse or dentist?*

I remember from such a young age, it was like I was always depressed and anxious. And I look back at photos when I was seven years old and I'm like, why was I fake smiling? Why did I need to fake-smile at the time, and also, why did I feel like I couldn't talk to somebody about why I didn't feel regular, why I was always angry, why I was always sad? In looking back, what I had to grow up with, those are natural emotions to have. But since I was hurting for so long, it's become pain, and it's pain that I still have to deal with.

Elders described how youth individually express pain as a result of a disruption of their culture, as well as how one is raised. An Elder described how she believed intergenerational trauma is linked to pain:

It is inherited like family pain and it's just never dealt with, so you just do the same thing, and that's where that term intergenerational trauma comes in, whereas you just don't deal with it and it's just always there, and everybody just kind of steps around it.

A clinician identified the role of the institution and optimism that pain is emerging in conversation so it can be dealt with sooner: “the recognition for it [pain] is a lot better than it ever used to be, because nobody really talked about it.” The three groups shared helpful strategies including, “ask youth how they think the pain interferes with their social interactions or every day life,” “keep that support open,” “be nonjudgmental,” “be compassionate,” “take different approaches working with them such as using harm reduction techniques,” “know the impact of colonization and institutionalization,” “listen,” “validate their perspectives,” and “engage with respectful body language.” The overall experience of pain as described by the three groups informed strategies that aid in individual healing and the important role of institutions in facilitating the process.

#### Share

Youth described mutual *sharing* of knowledge with clinicians as an opportunity for knowledge exchange and discussed their desire for a blend of Indigenous and Western practice when managing pain. Participants' recognized establishing clinical relationships by sharing information about one another provide comfort resulting in better health outcomes.

##### Sharing Knowledge About one Another

One youth shared how she faces challenges living in the city, yet despite this, recognized her role in achieving balance as an urban Indigenous youth: “Youth in the city must practice a certain type of wisdom to navigate living in the city and a connection with their culture.” Youth discussed helpful pain management strategies, including, teachings, sharing circles, connecting with Elders, traditional medicine, herbs, cedar, praying with tobacco, sweat lodge, smudging^*^^1^, art, drumming, singing, and dancing. Western remedies include physiotherapist, orthodontist, counseling, recreational activity, and pharmaceuticals.

##### Sharing Ways of Knowing

Clinicians faced their own challenges and experiences with navigating between Indigenous and Western practice. A clinician shared how she created a safe space as: “don't take the lead because then you empower them to help them make the decisions. I don't walk ahead, I don't walk behind, I walk with.” Another clinician discussed sharing vs. suppressing pain as she described her experiences understanding Indigenous pain: “no wonder it hurts, or no wonder people are suffering, right? And how do you get at those strengths, and how do you get us to really honestly, truly practice from a Medicine Wheel perspective as opposed to falling into how we were trained.” An Elder shared his perspective of pain as symbolizing water, whereas another Elder described sharing as, “when I deal with our people, I'm sharing about me too, because that's part of the relationship building. It's not just about what they're going to give. It's letting them know about you too.”

#### Teachable Moments

The fourth theme that emerged was the importance for youth to re-establish balance by capitalizing on teachable moments and creating spaces for youth to teach clinicians as well. Clinicians described their role in teaching youth about resources to help connect with culture and recognized the importance of consulting traditional healers. One clinician said: “Introducing them to the traditional healing department. When it comes to a lot of the traditional medicines, I always make sure to let them know that that's not my expertise area, but certainly there's things that we can offer here.” Another clinician described the importance of youth connecting with their culture:

They're disconnected from our teachings or to our way of being in that traditional sense, in that cultural sense, a way of being. There's that disconnect maybe from residential school, intergenerational trauma—Sixties Scoop, and so on and so forth, right? So, that all impacts a lot on how our families are today, that disconnect to all of that holistic piece. Because I know sometimes the families just don't know, because there's no one there to really teach them these aspects do matter, and it is important to your whole wellbeing.

Elders shared their concerns of Western medicine and the impact of youth misusing pharmaceutical medication. One Elder who worked at an Indigenous treatment centre shared her dilemma of choosing the right solution: “we cannot just give them medication because that's what they're asking for. And so, you have to teach them, to share that there's other ways of taking care of that health problem.” Another Elder described pain as: “It's one of our most powerful teachers. That's the way I've been taught. Because if we've got pain, we're not listening to our body. We're not taking care of ourselves.” Elders and clinicians recognized culture as treatment/therapy, however it is often disregarded and when found can be used as a health promoting tool to manage hurt and pain. Clinicians should offer teachable strategies from both an Indigenous and western perspective which may include, smudging, sweat lodge, going out for a walk/stairs, physical activity, nature and fresh air, mindfulness, water, medicine, and the environment.

#### Engage

The theme of engagement in the clinical setting was important as clinicians indicate they learn best from *listening* to their patients and using their own story in the way care is provided from a holistic perspective. One youth described the need for clinicians to share the clinical encounter with them and engage in pain conversations with an understanding of culture as she shared: “It's like the connection though. People need to feel like they mean something… it's the connection to their families, the connection to their communities, connection to the land.” Youth shared engagement strategies such as “educating them to know that it's okay to feel this way, and that it's normal to feel this way,” “encouraging them that we are the next generation, and that whatever we create and how we all work with each other now will benefit us all,” “describe what you're feeling right now, you can change that,” “be somebody in the world that can help how you're feeling right now,” and “to be inspired, is what the youth need. They just need to be inspired.”

Clinicians noted how youth state they don't feel heard, listened to or their concerns were not taken seriously. A clinician shared an engagement strategy may be offering the youth something to hold such as sweet grass or sage in the clinic. Another clinician discussed ways to engage in conversation with youth by initiating questions that will give them the power to direct the care they want to receive:

Asking them what they would like to see. Had they ever thought of these options? Give them options. Don't ask them, well, what do you want? What are the types of things you like to do? What works best for you? They may tell you. They may not. They may just sit there and listen for the first time. But if they come back, then you know you've done your job, right?

Elders recognized engagement as providing reassurance and supporting Indigenous youth with there own belongingness, hopes, dreams, goals, and desires:

You have this isolation from where you belong. So, I think there's intergenerational trauma, which I think that's definitely contributed too, as far as where colonialism has gone, but also just looking at it from a youth perspective, where they're really trying to figure out where they are, who they are, where they're going?

Experiences of poor engagement resulted in youth who reported that they do not feel listened to, are left wondering and do not have options to support them in how to cope with their painful experiences. Participants recognized that engaging in the historical, social and cultural components of pain facilitated feelings of confidence between youth and clinicians.

#### Navigate

Collectively, participants described how hard it is for youth to seek out help and the challenges they face to have their voices heard. This theme described the need for clinicians to help youth *navigate* and connect with relevant Indigenous and Western community services and resources. One youth described her frustration with a clinician offering a Western-based survey to assess her health concerns:

She was giving me these kind of surveys from one to 10, and it was just paper and it was basically telling me to list all of the reasons that I wanted to kill myself, and it was asking me how bad I wanted to kill myself, and all this stuff. It just wasn't helping.

This approach within clinical care was also raised by Elders, who discussed the importance of clinicians seeking out options that offer the appropriate support at the right time for youth, and the need for follow up appointments to reassess, build clinical trust and strengthen relationships. Clinicians recognized the importance of advocating for youth with other colleagues while supporting youth to navigate what is working and not working. Another clinician shared “I think from our positions, well what we're trying to do is direct them to, especially Aboriginal or Indigenous youth to connect with their culture, that there is counseling available. There are people that care, that kind of thing.”

Ensuring that the individualized needs of youth are addressed with appropriate and culturally safe support were identified as being crucial in helping youth reconnect with their community and validate the important role of institutions. Furthermore, Indigenous navigation services aided in the development and guidance for youth to navigate a support system similar to a traditional family unit that would infuse cultural knowledge and ways of staying healthy using traditional practices. Youth identified that the positive ways they manage their pain is through songs, being in nature, smudging, spending time with Elders and being with others.

## Discussion: Striving for Connections to Balance Health and Reduce Pain

In this study 33 Elders, clinicians and youth identify consistent themes relating to the dimensions of hurt and pain and the practical ways for clinical intervention considering the Medicine Wheel and Two-Eyed Seeing lens. There is an underlying theme of the importance of connectedness, kinship and relationships for maintaining health. The Medicine Wheel guides the symbolic balance and interconnection between physical, spiritual, emotional and mental health. When considering the notion that health is a balance of the dimensions of the Medicine Wheel three main thematic areas emerged across the three groups and include (1) predictors of imbalance; (2) indicators of imbalance; and (3) strategies to re-establish balance. The main strategy includes the LISTEN (*Language, Individual, Share, Teachable moments, Engage, and Navigate*) approach that outlines cultural considerations and a safe guide for clinicians to assist with identifying and managing pain and hurt. The results further highlight that the concept of pain and hurt may be experienced and defined differently by Indigenous people which may explain why it remains underassessed and undertreated.

Findings from previous studies note pain is an unrecognized concept with no word directly translating to pain in First Nations languages (8, 31). The experience of pain has also been described using the dimensions of the Medicine Wheel and from a historical, community and family perspective consistent with the findings of the current research. For example, in research with First Nation communities in Mi'kma'ki (1) c*ultural* and *spiritual* pain were described as ‘loss of language', cutting of hair, and centralization and relocation of communities. There was a sense of ‘*community* hurt' and hurt between people “you got hurt or someone hurt you” and these comments were similar by this study. The original study was conducted in rural First Nation communities and the current study was with urban participants highlighting that irrespective of place of residence; the experience, dimensions and scope of pain and hurt may be similar for urban and rural Indigenous youth. This study's findings are consistent with other research where clinicians identified the importance of connection and relationships in Indigenous youth for balancing wellness-such as share, exchange through teachable moments, engage and support for navigation. These findings are aligned with the FIRST principles which are clinician guidelines originating from a similar study with Indigenous children and youth in four First Nations communities in Mi'kma'ki. FIRST principles (1) support clinicians to understand who *family is*, how *information* is exchanged, the importance of building *relationships*, creating *safe spaces* and considering *treatment* from both an Indigenous and western lens when supporting Indigenous youth. In work related to creating humanizing health spaces for Indigenous People, Indigenous researcher Sylliboy and Hovey (32) share the idea that, nurturing positive relationships encourages trust with clinicians who are not familiar Indigenous People as there is a vulnerability for patients in these settings. Health clinicians need to find ways to learn, trust and care (32).

Culture is not a stationary concept, it is shaped by social construct, environment, relationships, beliefs, values and according to Matheson et al. (33) “rooted in ancestry (systems of knowledge), historical events (collective trauma), and evolving contexts (climate change, colonization, migration)” (p. 3). The dimensions of language related to culture, health, body language, expression and information exchange highlight the continuing importance of context of care. Previous research suggests that pain may be withheld to avoid attention and often seen as stoicism as a result of pain being viewed as a weakness in Indigenous cultures (6, 8). Clinicians are trained to use numerical or faces pain scales, however, only minimal research has been done to determine if these self-report pain scales are culturally appropriate for use with Indigenous youth (34, 35) and there is evidence that they are not helpful to First Nations youth (10) which was confirmed again in this research. Having language speakers with health care knowledge has been a recommendation to improve the navigation of the health care journey (1, 36, 37).

Striving to provide *individualized* care that holistically considers the strengths and unique needs of each individual without making assumptions about who they are or what they need is consistent with previous research (10). Similar to themes in the current study, Browne et al.'s (38) research with two urban Aboriginal Health Centres identified four key dimensions of equity-oriented health services including inequity-responsive care, culturally safe care, violence-informed care and contextually tailored or individual care. Individualizing care is a priority due to the evidence that Indigenous youth conceptualize, and experience pain differently compared to non-Indigenous youth consequently putting them at higher risk for poorer outcomes (1, 4). Providing youth with the opportunity to hold something that is culturally significant to them in the clinical setting is a tangible strategy easily implemented and step toward respectful practice and relationship building. Relationship building and meaningful engagement has been reported before as an essential component to the health journey of Indigenous people (39, 40).

Youth in this study reported accessing clinicians as a usual source of care to consult or receive medical advice and responded they felt comfortable seeking care yet there were some accepted practices in the mainstream system that may cause harm. There is a broader need for clinicians to take responsibility to educate themselves about Indigenous health, colonization and its impacts on health while reflecting on their own assumptions/biases and how these can perpetuate all dimensions of pain (emotional, spiritual, physical, mental). Evidence has shown that a connection to culture can buffer the predictors of imbalance and potentially reduce the experience related to the indicators of imbalance (1, 10, 33, 41). A large scoping review identified that culturally safe strategies can decolonize care by demonstrating awareness of colonialism, racism, and discrimination. The review identified strategies that build partnerships and share decision-making in the delivery of care all contribute to the philosophy of the LISTEN approach (42). A study by Jacklin et al. (37) with five Indigenous communities, reports that health care relationships are central to addressing the ongoing colonial dynamics in Indigenous health care and support the notion that they play a role in buffering past harms.

## Limitations

One limitation of this study is the method used to collect data did not guarantee anonymity when participants selected conversation sessions vs. one-on-one interviews. This may have resulted in participants sharing perspectives to their personal agency of safety. Another limitation included the notion that engaging more communities could have provided a broader range of community member experiences. In addition, providing the participants an opportunity to speak in their own language would have potentially given more information about the role of language in the concept of pain.

## Conclusion

The findings of this study have been interpreted by community members and health leaders, providing clinicians with the evidence to provide strengths-based clinical experiences and culturally safe care for Indigenous youth. The findings provide specific clinical implications to advance health clinicians knowledge and understanding to provide culturally safe care. Being culturally safe means knowing the history and the impact of that history on Indigenous People's health. Clinicians can consider those indicators of imbalance and how they can support indigenous people to re-gain balance using both Indigenous and Western ways of knowing and doing. Guided by the LISTEN approach clinicians can keep the idea of culturally safe care front of mind, and this approach can make healing feel achievable. The LISTEN approach will enrich cultural safety curriculum, integrate Indigenous Knowledge for clinicians, and promote the likelihood of healing.

## Data Availability Statement

The datasets presented in this article are not readily available because all data generated from this study belongs to the Community Health Centre as proxy owners for the study participants, as dictated by OCAP principles. Requests to access the datasets should be forwarded to ANaveau@dahac.ca.

## Ethics Statement

The studies involving human participants were reviewed and approved by IWK Health Centre Research Ethics Board and Mi'kmaq Ethicswatch. The participants provided their written informed consent to participate in this study.

## Author Contributions

RV involved in design, data collection, data analysis, and writing of manuscript. ML and AN involved in design, data analysis, and writing of manuscript. All authors contributed to the article and approved the submitted version.

## Conflict of Interest

The authors declare that the research was conducted in the absence of any commercial or financial relationships that could be construed as a potential conflict of interest.

## Publisher's Note

All claims expressed in this article are solely those of the authors and do not necessarily represent those of their affiliated organizations, or those of the publisher, the editors and the reviewers. Any product that may be evaluated in this article, or claim that may be made by its manufacturer, is not guaranteed or endorsed by the publisher.

## Footnotes

^1^Smudging is a tradition, common to many First Nations, which involves the burning of one or more medicines gathered from the earth. The four sacred medicines are tobacco, sage, cedar, and sweetgrass. The most common medicines used in a smudge are sweetgrass, sage, and cedar. There are many ways to perform a smudge and different variations exist. Smudging the whole being is meant to portray only the good part of our self through actions. Smudging allows people to stop, slow down, and become mindful and centred. This allows people to remember, connect, and be grounded in the event, task, or purpose at hand. Smudging also allows people to let go of negative feelings and thoughts. Letting go of things that inhibit a person from being balanced and focused comes from the feeling of being calm and safe while smudging (30).
